# Synergistic interaction between galectin-3 and carcinoembryonic antigen promotes colorectal cancer metastasis

**DOI:** 10.18632/oncotarget.18721

**Published:** 2017-06-27

**Authors:** Keng-Liang Wu, Eng-Yen Huang, Wen-Ling Yeh, Chang-Chun Hsiao, Chung-Mou Kuo

**Affiliations:** ^1^ Division of Hepatogastroenterology, Department of Internal Medicine, Kaohsiung Chang Gung Memorial Hospital, Kaohsiung, Taiwan; ^2^ Graduate Institute of Clinical Medical Sciences, Chang Gung University, Kaohsiung, Taiwan; ^3^ Chang Gung University, College of Medicine, Kaohsiung, Taiwan; ^4^ Department of Radiation Oncology, Kaohsiung Chang Gung Memorial Hospital, Kaohsiung, Taiwan; ^5^ School of Traditional Chinese Medicine, Chang Gung University College of Medicine, Kaohsiung, Taiwan; ^6^ Center for Shockwave Medicine and Tissue Engineering, Kaohsiung Chang Gung Memorial Hospital, Kaohsiung, Taiwan

**Keywords:** colorectal cancer, galectin-3, carcinoembryonic antigen, cancer cell migration

## Abstract

In this study, we investigated the role of galectin-3 and carcinoembryonic antigen (CEA) in metastasis and survival of colorectal cancer (CRC) patients. CEA interacted with galectin-3 at the cell surface and cytoplasm of Caco2 and DLD1 CRC cells. Knocking down galectin-3 did not affect CEA expression in CRC cells. However, there was a dose-dependent increase in CRC cell migration upon addition of small amounts of exogenous CEA (≤1ng/ml). Galectin-3 knockdown blocked induction of CRC cell migration by CEA, suggesting interaction between galectin-3 and CEA was necessary for CRC cell migration. Exogenous CEA and galectin-3 synergistically promoted migration of galectin-3 knockdown DLD1 cells. Immunohistochemical analysis showed that CEA co-localized with galectin-3 in CRC patient tissues. In additon, advanced stage CRC patients had higher serum galectin-3 and CEA levels than early stage CRC patients. High serum CEA and galectin-3 levels correlated with advanced N stage and poor survival in CRC patients. These findings suggest interaction between galectin-3 and CEA promotes CRC migration and metastasis, and correlates with poor survival of CRC patients. Thus combinatorial therapy targeting galectin-3 and CEA may improve outcomes for advanced stage CRC patients.

## INTRODUCTION

Colorectal cancer (CRC) is one of the most common cancers and a principal cause of death worldwide. The morbidity and mortality rates of CRC continue to increase because CRC is diagnosed at advanced stages in most cases resulting in poor prognosis. The rate of local recurrence and metastasis of CRC is 25% to 50% [1, 2]. The 5-year risk of systemic recurrence in CRC patients after surgical resection is approximately 50% for those with lymph node involvement and 20% to 30% if lymph nodes are negative [2]. Relapses often occur in the liver, focal colon, lung ovaries, bone, anastomosis or brain.

Metastasis is a multistage process by which cancer cells escape from the primary cancer and establish secondary foci at distant sites. The development and progression of many malignancies including colorectal cancer are associated with aberrant activation of multiple signaling pathways that promote migration, proliferation and survival of tumor cells resulting in metastasis [3]. Our previous study demonstrated that overexpression of galectin-3 increased colon cancer cell migration by activating the K-Ras-Raf-ERK pathway [4].

Galectin-3 (also known as Mac-2, CBP-30, hL-31, CBP-35, IgEBP, and LBL) is an endogenous β-galactoside-binding protein belonging to a family of widely distributed carbohydrate binding proteins that are implicated in cell differentiation, adhesion, and malignant transformation. Galectin-3 is involved in the transformation and metastasis of human tumors such as prostate, breast, ovarian, lung, thyroid and pancreas cancers [5–8]. High expression of galectin-3 is correlated with the malignant behavior [9] and metastasis [10, 11] of human colon cancer cells. Galectin-3 regulates metastasis by binding to cell adhesion-related molecules and inhibiting cell–cell and cell–matrix interactions [12], thereby augmenting cancer cell detachment and mobility [13, 14]. Although extracellular expression of galectin-3 modulates the various stages of metastasis in human colon cancer, the mechanistic details of its role in intracellular signaling, migration, and metastatic potential of colon cancer cells are unclear.

Carcinoembryonic antigen (CEA, CEACAM5, and CD66e) is expressed on many tumors [15, 16] and is associated with malignancy, particularly enhanced metastasis [17–19]. It functions as a chemoattractant and as an adhesion molecule [20] and promotes metastasis in some tumors [21, 22]. The interaction between galectin-3 and CEA is not clear. Galectin-3 has been shown to bind to human CEA, and lysosome-associated membrane glycoproteins that are involved in cell adhesion [23–25]. Galectin-3 also co-localizes with CEA on the KM12 cell surface [26].

Therefore, we explored the association between galectin-3 and CEA and their potential role in metastasis of colon cancer in this study.

## RESULTS

### Galectin-3 and CEA expression on colon cancer cells

**Figure 1:**
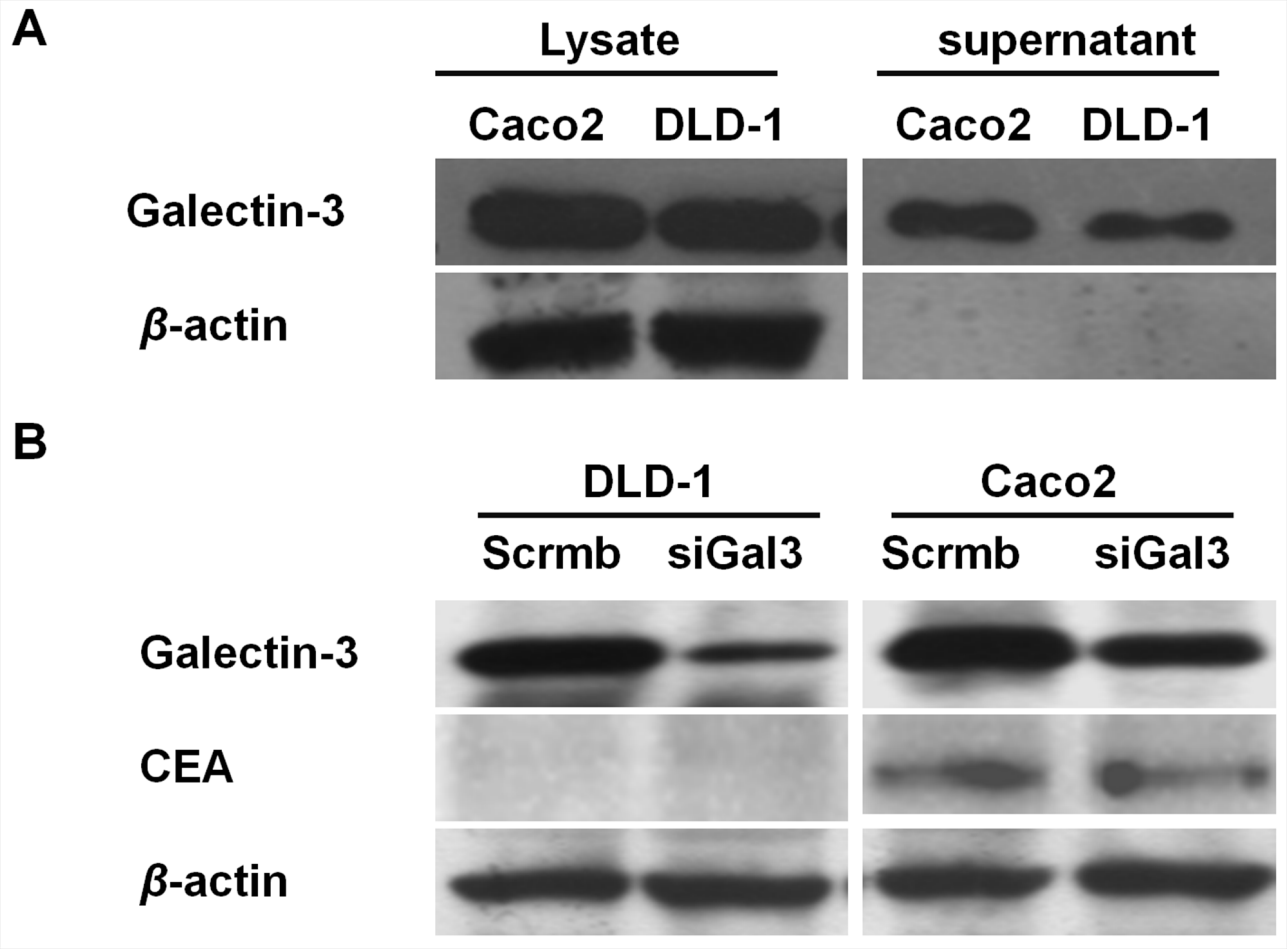
Galectin-3 and CEA expression in colon cancer cells **(A)** Western blot analysis shows galectin-3 levels in total cell lysates and supernatant of Caco2 and DLD-1 colon cancer cells. β-actin was used as loading control.Galectin-3 expression is higher in Caco2 lysates and supernatant compared to DLD-1. **(B)** Western blot analysis shows CEA and galectin-3 levels in DLD-1 and Caco-2 cells transfected with scramble or gal-3 siRNAs. β-actin was used as loading control.CEA levels were higher in Caco2 cells compared to DLD-1cells. Galectin-3 knockdown does not effect CEA expression.

Western blot analysis revealed that galectin-3 was expressed in both cell lysates and supernatants of DLD1 and Caco2 colon cancer cells (Figure 1A). Galectin-3 expression was higher in Caco2 cells compared to DLD-1 (Figure 1A). Higher CEA expression was also observed in Caco2 cells compared to DLD-1 cells (Figure 1B). Moreover, siRNA knockdown of galectin-3 had no effect on CEA expression in both DLD-1 and Caco2 cell lines (Figure 1B).

### Interaction between galectin-3 and CEA in colorectal cancer cells

**Figure 2:**
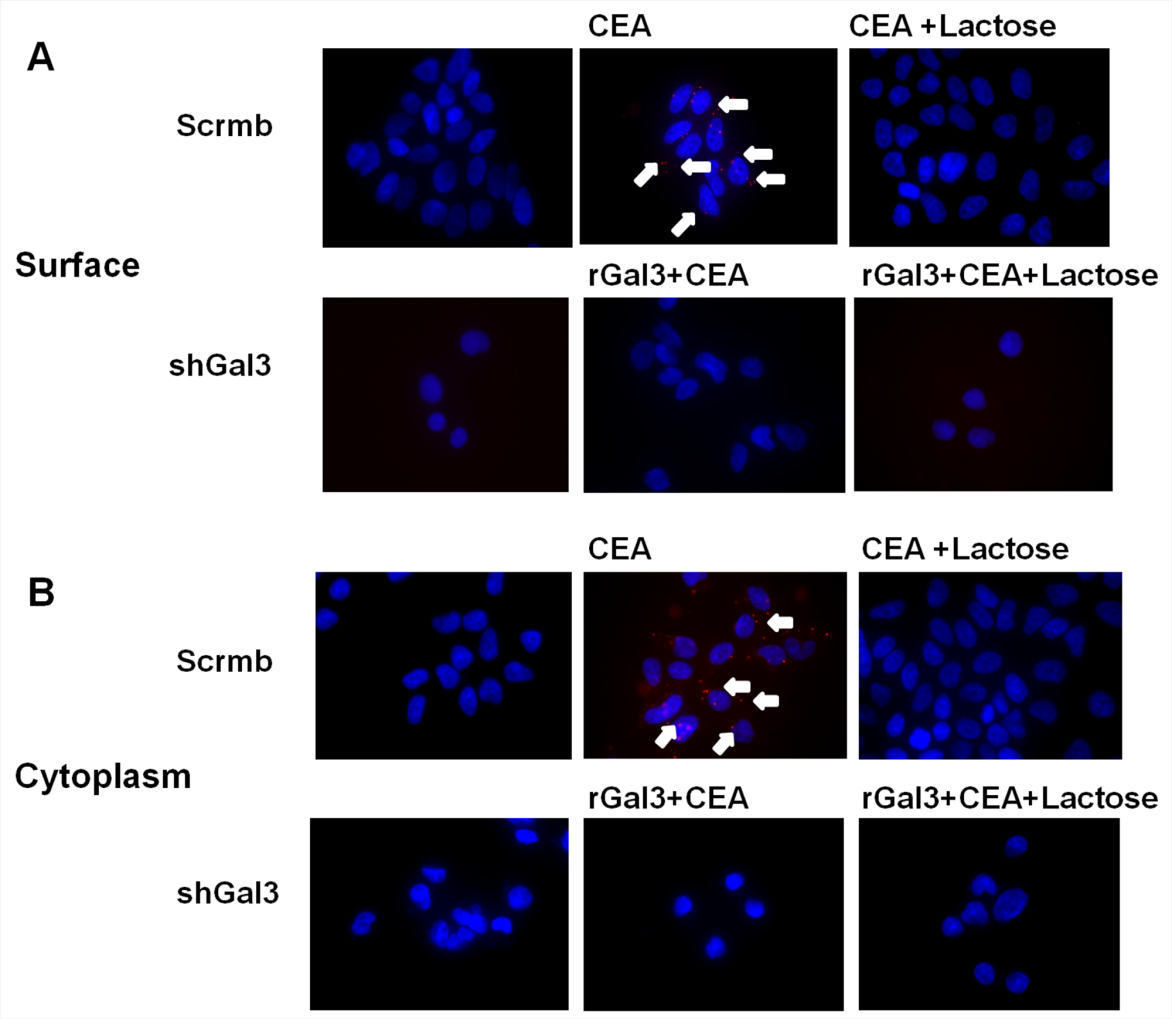
PLA analysis of interaction between galectin-3 and CEA Interaction between galectin-3 and CEA on **(A)** cell surface and **(B)** cytoplasm of colorectal cancer cells (DLD-1) is determined by PLA method. The reaction was blocked by 30mM lactose. CEA (1ng/ml) did not interact with rGal3 (30μg/ml) on cell membrane or in cytoplasm of colorectal cancer cells. The scramble and gal-3 shRNA transfected CRC cells were analyzed to demonstrate the bonafide interactions.

Next, we performed proximity ligation assay (PLA) and found that galectin-3 and CEA interacted with each other at the cell surface (Figure 2A) as well as in the cytoplasm of colorectal cancer cells (Figure 2B). This interaction was blocked by lactose. Interestingly, CEA did not interact with recombinant galectin-3 (rGal3) on the membrane surface and in the cytoplasm (Figure 2A, 2B).

### Exogenous CEA and galectin-3 synergistically enhance migration of galectin-3 knockdown DLD-1cells

**Figure 3:**
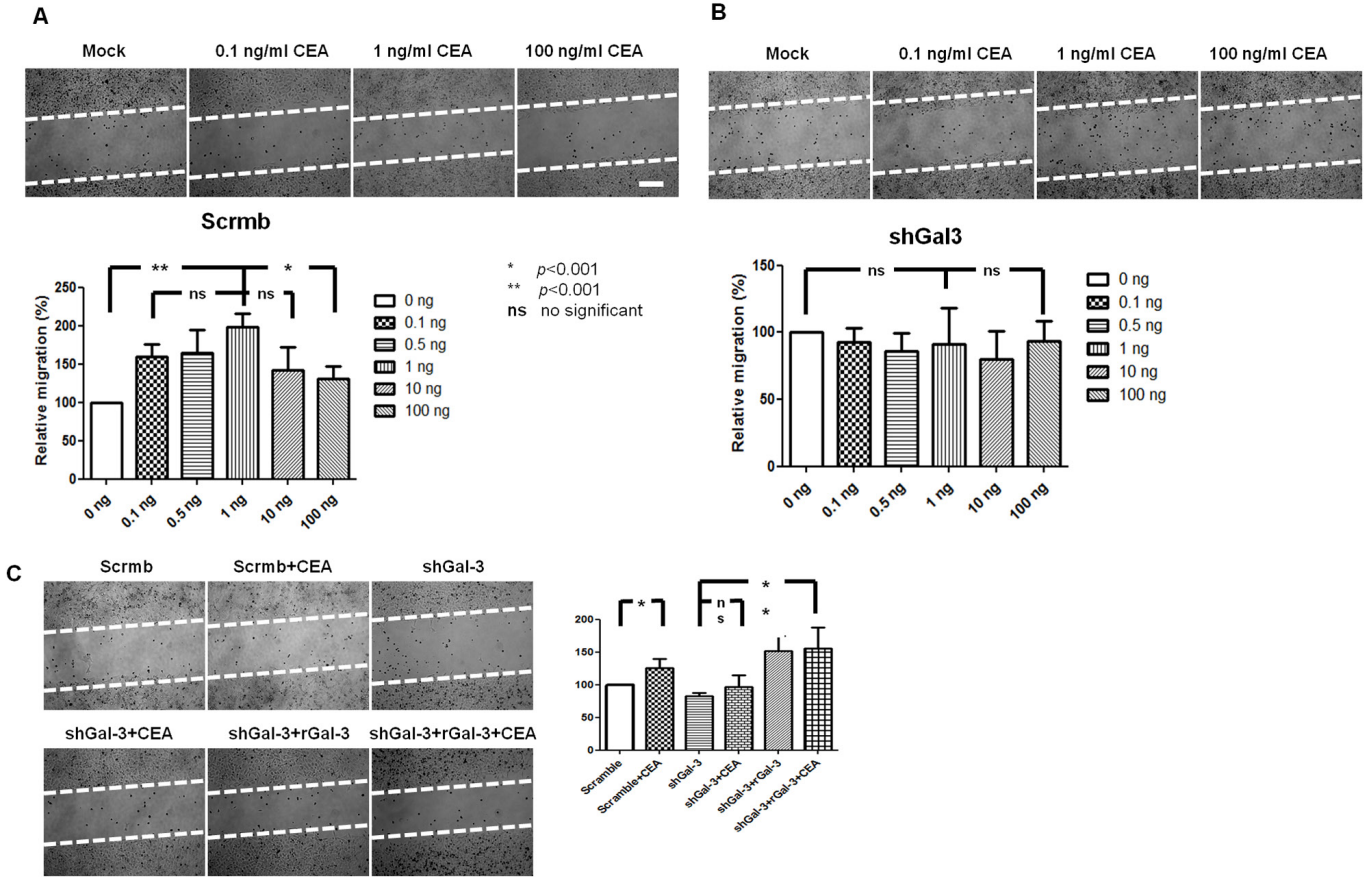
Effect of exogenous CEA and galectin-3 on *in vitro* DLD-1 cell migration **(A)** Representative images show increased migration of DLD-1 with 0.1, 0.5 and to 1ng/ml CEA and decreased migration with 10 and 100ng/ml CEA. **(B)** Representative images show no effect of different CEA concentration on migration of galectin-3 knockdown DLD-1 cells. **(C)** Synergistic effect of CEA (1ng/ml) and rGal3 (30ug/ml) upon migration of shRNA Gal3 DLD-1cells. Note: Migration was determined by wound healing assay. White bar denotes 100μm. * : *p*<0.001; ** : *p*<0.001; ns : no significant.

Next, we observed that 1ng/ml CEAincreased the migration of DLD-1 cells that were transfected with scrambled shRNA, but had no effect on DLD-1 cells transfected with shRNA against galectin-3 (Figure 3A, 3B). We observed a dose-dependent effect on DLD-1 migration for doses ≤1ng/ml CEA, whereas CEA doses above 1ng/ml decreased migration. Additionally, we observed that addition of recombinant galectin-3 enhanced the migration of shRNA Gal3 DLD-1, thereby demonstrating synergy between galectin-3 and CEA (Figure 3C).

### Galectin-3 and CEA expression correlates with advanced stage on CRC

**Figure 4:**
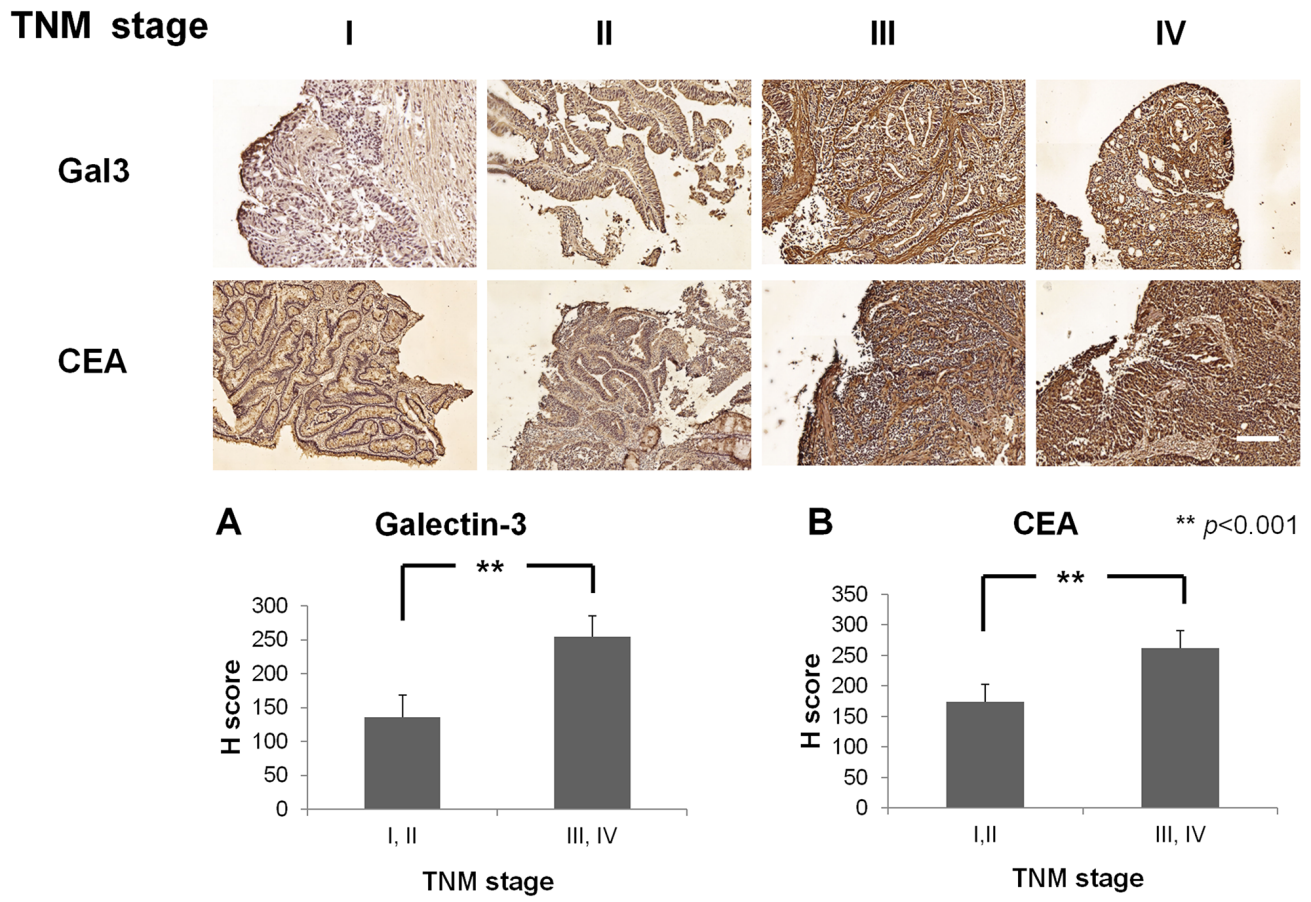
Immunohistochemical staining of CRC patient samples with Gal3 and CEA Representative images of CRC patient samples of TNM stages I,II,III and IV immunohistochemically stained with anti-Gal3 antibody (top panel) and anti-CEA antibody (bottom panel). Quantitative plots show H-score analysis of Gal-3 and CEA expression based on TNM stages I&II and II&IV. Fourteen CRC patients (TNM stage I:2; II:5; III:5; IV:2) were analyzed. Higher expression of galectin-3 (H score, III, IV: I, II: 254±31: 136±33, *p*<0.001) and CEA (H score, III, IV: I, II: 261±28: 174±28, *p*<0.001) was observed in advanced stage (III, IV) compared to early stages (I, II). White bar represents 100μm.

IHC staining in 14 patient samples representing different TNM stages (I:2; II:5; III:5; IV:2) showed increased expression of galectin-3 in advanced stages III&IV compared to early stages I&II (H score, III&IV: I&II: 254±31: 136±33, *p*<0.001; Figure 4A). Similarly, CEA was highly expressed in advanced stages compared to early stages (H score, III&IV: I&II: 261±28: 174±28, *p*<0.001; Figure 4B). Thus, galectin-3 and CEA expression correlated with advanced stage CRC.

### Serum galectin-3 and CEA expression in CRC patients correlates with N stage and patient survival

**Figure 5:**
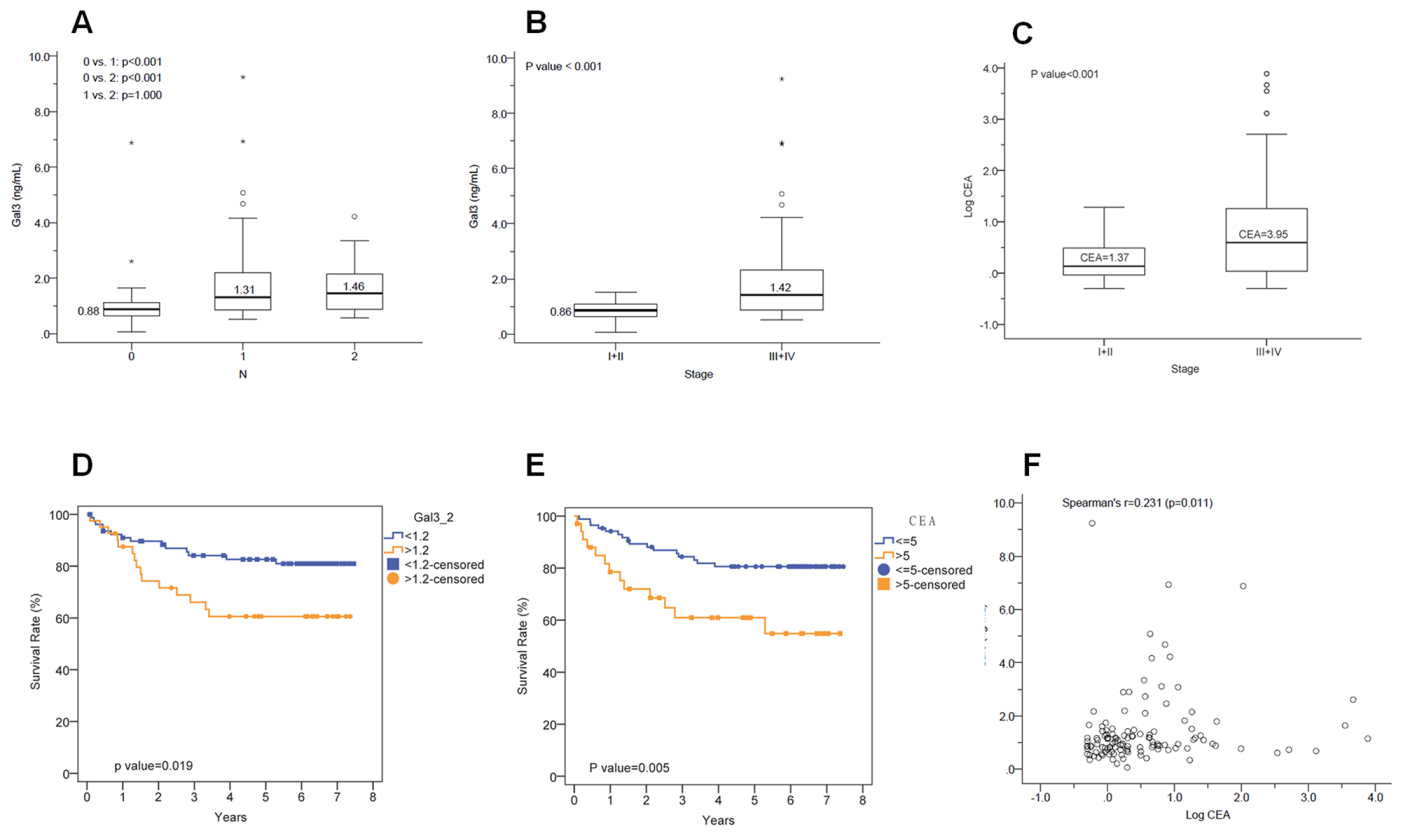
Correlation between Gal-3 and CEA levels with N stage and survival **(A)** Histogram shows galectin-3 expression levels in N stages (lymph nodule) 0, 1 and 2. There were significant differences between N (lymph nodule stage) with galectin-3 expression (N1, N2 : N0, *p*<0.001). **(B)** Histogram plot shows galectin-3 expression in TNM stages I&II and III&IV. High galectin-3 expression is observed in advanced stages (III, IV) of colorectal cancer than early stages (I, II) (early stage: advanced stage, *p*<0.001). **(C)** Histogram plot shows CEA expression in TNM stages I&II and III&IV. CEA expression is higher in advanced stages (III, IV) of colorectal cancer than early stages (I, II) (early stage: advanced stage, *p*<0.001). **(D)** Kaplan-Meier survival curves of CRC patients with low galectin-3 levels (blue; <1.2ng/ml) versus high galectin-3 levels (red; ≥1.2ng/ml). The survival rate was poor for patients with high galectin-3 compared to those with low galectin-3 (*p*=0.019). **(E)** Kaplan-Meier survival curves of CRC patients with low CEA levels (blue; <5ng/ml) versus high CEA levels (red; ≥5ng/ml). The survival rate was poor for patients with high galectin-3 compared to those with low galectin-3 (*p*=0.005). **(F)** Correlation analysis between serum galectin-3 and CEA levels (Spearman’s *r*: 0.231, *p*<0.05).

We analyzed 120 patients with different TNM stages of CRC (TNM stage I: 21; II: 39; III: 40; IV: 20) and observed significant differences between N stage (lymph nodule stage) and serum galectin-3 levels (Figure 5A). Advanced stage CRC correlated with high galectin-3 levels (Figure 5B).We also observed that higher serum CEA levels correlated with higher galectin-3 expression in advanced stage (III, IV) compared to early stage (I, II) CRC (Figure 5C). Kaplan-Meier survival analysis demonstrated that CRC patients with higher serum galectin-3 levels (≥1.2ng/ml) were associated with lower survival than patients with lower serum galectin-3 levels (<1.2ng/ml) (Figure 5D). Similarly, CRC patients with lower survival rate were associated with higher serum CEA levels (≥5ng/ml) compared to patients with lower serum CEA levels (<5ng/ml) (Figure 5E). Therefore, there was linear correlation between galectin-3 and CEA expression levels in CRC patients (Figure 5F).

## DISCUSSION

In this study, we demonstrated that extracellular CEA interacted with galectin-3 and promoted migration of colorectal cancer cells and distal metastasis. Boscher *et al.* clarified galectin-3/phospho-caveolin-1/RhoA signaling that mediates integrin signaling downstream, leading to matrix remodeling and tumor cell migration in metastatic cancer cells [13]. Saeland *et al.* demonstrated that CEA was recognized and taken up by antigen presenting cells with increased mannose expression and branched N-glycans, which correlated with human galectin-3 binding [23]. The individual tumor antigens contain distinct glycan structures that are associated with cellular interactions in a tumor microenvironment that may be consequential for cancer progression.

We observed a dose-dependent effect of CEA upon migration of DLD-1 colorectal cells with a dose-dependent increase below 1ng/ml and decreased migration above 1ng/ml. Jin *et al.* also showed that CEA promoted the migration of colorectal cells *in vitro* [27]. We postulate that CEA in excess of 1ng/ml may result in intercellular binding of CEA to galectin-3 on tumor cells, thereby inhibiting cell migration. However, this needs to be experimentally proven.

We also demonstrated that knockdown of Gal3 in DLD-1 cells resulted in abrogating their migration upon addition of 1ng/ml CEA. This suggested the specificity of the Gal3-CEA interaction in promoting CRC migration. Galectin-3 has a high affinity for glycoconjugates containing β-galactosides, especially polylactosamines. CEA is an 180±200 kDa glycoprotein and a member of the immunoglobulin super family of proteins with over 50% of its weight contributed by the carbohydrates. We postulate that galectin-3 recognizes the polylactosamine chains in CEA. Although there was no correlation between serum galectin-3 levels and TNM stage clinically, we observed its correlation with N and advanced stages. Shimura *et al* showed that the correlation between galectin-3 and TNM stage was one of the key factors in the regulating immunological, inflammatory and nutritional factors in CRC [28]. In the present study, the patient survival was correlated with high serum galectin-3 levels (≥1.2ng/ml). Further, we observed synergistic correlation between serum galectin-3 and CEA levels with different stages of colorectal cancer patients.

Both CEA and galectin-3 are multifunctional molecules. Saeland *et al.* first demonstrated the cell surface interaction between galectin-3 and CEA in human colon carcinoma cell lines [23]. Therefore, it was suggested that this lectin may have a role in colon carcinoma cell adhesion and tumor progression. We demonstrated that the interaction between galectin-3 and CEA molecules was based on their expression. Further investigations are necessary to understand the nature of their interaction. We also observed that blocking the interaction between galectin-3 with CEA inhibited colon cancer cells migration. This suggests that therapeutic targeting of galectin-3 and CEA may have a synergistic effect on distal metastasis of colorectal cancer.

## MATERIALS AND METHODS

### Cell lines

The human colon cancer cell lines, DLD-1(BCRC60132) and Caco2(BCRC60182) were derived from a well-differentiated colonic adenocarcinoma and characterized extensively. Cell lines were grown in Dulbecco’s modified Eagle medium (Gibco/ BRL, Rockville, MD) supplemented with 10% fetal bovine serum, penicillin (100U/ml), and streptomycin (100g/ml). The cell lines transfected with sense or antisense galectin-3 constructs were generated as previously described [4].

### Preparation of human Gal-3

Human galectins were prepared as outlined previously. The recombinant galectin-3 was purified by affinity chromatography on lactosyl-Sepharose, and the bound lectin was eluted with 100mM lactose in PBS and 14mM β-mercaptoethanol (β-ME). Prior to derivatization, β-ME was removed from the galectin-3 samples by passing through a PD-10 gel filtration column (GE Healthcare), followed by the addition of 100mM lactose to help maintain the stability of galectin-3 and reduce the likelihood of adduct formation at or near the carbohydrate recognition domain. Alexa Fluor 488-labeled galectin-3 was prepared with either Alexa Fluor 488 C5-maleimide or Alexa Fluor 488 carboxylic acid succinimidyl ester dilithium salt-reactive dyes (Molecular Probes) according to manufacturer’s protocol.

### SiRNA knockdown of galectin-3

The siRNAs for silencing galectin-3 and the scrambled negative control siRNA were obtained from Invitrogen (StealthRNAi 1299001,USA). Transfection was performed as described by Wu *et al* [4]. Briefly, 7.5μl siRNA was mixed with 250μl Opti-MEM. Concurrently, 7.5μl lipofectamine^TM^ 2000 and 250μl Opti-MEM were mixed and incubated for 5 min. The diluted siRNA and lipofectamine mixtures were mixed for 20 min and added to 6-well plates in which DLD-1 cells had been seeded (5-9x10^5^ cells/well) for 4h. Control cells were treated with StealthTM RNAi negative control duplex (Invitrogen).

### Galectin-3 shRNA transfection

The shRNA for human galectin-3 mRNA (Clone TRCN0000029305) was designed and purchased from National RNAi Core Facility (Taipei, Taiwan). The pLKO.1 plasmid containing Galectin-3 shRNA, psPAX2 packaging plasmid (Addgene.com, Cambridge, MA, USA), and pMD2.G envelope plasmid (http://Addgene.com, USA) were co-transfected into HEK-293T cells with FuGENE6 Transfection Reagent (Roche Applied Biosciences, Penzberg, Germany). Viral particles were collected from the medium and stored at -80 °C. For lentivirus infection, 1x10^6^DLD-1 cells were seeded in a 25T flask and grown to approximately 70% confluence. The cells were infected with either scrambled shRNA lentivirus or pLKO.1 Gal-3 shRNA lentivirus for 24h in polybrene (Sigma-Aldrich, St. Louis, MO, USA) containing medium. Then, fresh puromycin (Sigma- Aldrich, St. Louis, MO, USA) containing medium was added everyday for selecting infected cells. Western blot analysis was used to detect Galectin-3 protein expression after selection of stably transfected DLD-1 cells.

### Western blotting

The proteins (20 μg) extracted from wild-type DLD-1, Caco2 and knockdown of Gal3 in ng DLD-1 cells were subjected to 12% SDS-PAGE at (90 ) voltage for 100 min electrophoresis followed by transfer onto a PVDF membrane (Millipore, Billerica, MA, USA) at (25 ) voltage for 60 min. Then, the membranes were blocking with 5% skimmed milk and incubated with primary antibodies overnight at 4°C followed by incubation with secondary antibodies. The protein bands were detected with Western Lightning Plus–ECL, Enhanced Chemiluminescence Substrate (PerkinElmer Inc, Waltham, MA, USA). Membranes were then stripped and re-probed with antibodies against housekeeping genes (as internal controls) and developed with ECL. The primary antibodies against galectin-3, and CEA were all obtained from Cell signaling technologies, Beverly, MA, USA.

### Tumor cell migration assay

8x10^5^/ml DLD-1 cells (Scramble and shGal3) were cultured in the 500μm wilde culture insert (ibidi, Germany) absence of serum and remove the insert after 24h incubation. The wounded monolayers were washed twice to remove non-adherent cells. After washing with PBS, the wounded regions were allowed to heal for 24h in serum free medium prior to analysis. The migration assay was quantified as the ratio of the remaining cell-free area to the area of the initial wound (calculated as a mean percentage) using the public domain software Image J (http://rsbweb.nih.gov).

### *In situ* proximity ligation assay (PLA)

The interaction between Gal-3 and CEA was determined by the Duolink reagent kit (Olink Biosciences, Uppsala, Sweden). A total of 1x10^3^ cells were seeded onto a chamber slide. Cells were fixed in 1% paraformaldehyde and washed with wash buffer A. Cells were then incubated with blocking solution at 37°C for 30 min followed by incubation with primary antibodies, mouse anti Galectin-3 (Santa Cruz) and rabbit anti CEA (Santa Cruz) at 37°C for 60 min. The cells were further washed and incubated with th corresponding PLA probe (anti mouse PLUS and anti rabbit MINUS) at 37°C for 60 min. The probes were then ligated with ligation solution and amplified with amplification solution. The slides were imaged by fluorescence microscopy (Axio Observer Z1, Carl Zeiss MicroImaging, Inc., Welwyn Garden City, UK).

### Immunohistochemistry of galectin-3 and CEA in colon cancer tissue sections

This study protocol was approved by the Institutional Review Board of the Kaohsiung Chang Gung Memorial hospital. Immunohistochemistry for galectin-3 and CEA expression in the colon cancer tissues of patients was performed using a Vectastain ABC kit and a DAB substrate kit (Vector Laboratories, Burlingame, CA, USA) according to the manufacturers’ instructions. Fourteen colon cancer tissues of different stages (TNM stage I, II, III and IV) were studied. The sample size was chosen based on a study power of 0.8, 2-sided alpha level of 0.05, and 50% higher expression of galectin-3 in advanced colon cancer tissues. The H-score method was adopted to assign a score to each patient based on the percentage of cells at different staining intensities. The staining intensity was scored according to four categories: 0, no staining; 1+, weak staining; 2+, moderate staining; and 3+, strong staining. H-score was calculated using the formula: 1× (percentage of 1+ staining) + 2× (percentage of 2+ staining) + 3x (percentage of 3+ staining) and ranged from 0 to 300.

### ELISA for serum galectin-3 and CEA

The 96-well plates were coated with 2.5mg/ml anti-galectin antibody in coating buffer (15mM Na_2_CO_3_ and 17mM NaHCO_3_, pH: 9.6) overnight at 4°C.The plates were washed with a washing buffer (0.05% Tween-20 in PBS) and incubated with blocking buffer (1% bovine serum albumin in PBS) for 1h at room temperature. Serum samples or standard recombinant galectins were added to the plates for 2h before application of biotinylated anti-galectin antibody (1.25mg/ml in blocking buffer) for 1h at room temperature. After addition of ExtrAvidin peroxidase (1:10,000 dilution in blocking buffer) for 1h, the plates were developed with Sigma FAST OPD for 10 minutes. The reaction was stopped by adding 4mM sulfuric acid, and the absorbance was read at 492nm by a microplate reader. We measured the exact concentration of CEA (R&D), and galectin-3 (R&D) by intrapolation of a standard curve made by a series of known concentrations as per manufacturer’s instruction.

### Statistical analysis

Data were expressed as mean ± SD values. Statistical analysis was performed either by one-way analysis of variance and subsequent Tukey multiple comparison tests or by two-way analysis of variance with subsequent Bonferroni post-tests. Contingency table analysis and χ^2^ tests were used to study the relationship between tumor stage results and morphological parameters. All of these were performed using the SPSS Software (version 13). *p* < 0.05 was considered statistically significant.
